# Investigation of the effect of dietary intake of omega‐3 polyunsaturated fatty acids on trauma‐induced white matter injury with quantitative diffusion MRI in mice

**DOI:** 10.1002/jnr.24705

**Published:** 2020-08-25

**Authors:** Laura D. Reyes, Thaddeus Haight, Abhishek Desai, Huazhen Chen, Asamoah Bosomtwi, Alexandru Korotcov, Bernard Dardzinski, Hee‐Yong Kim, Carlo Pierpaoli

**Affiliations:** ^1^ Quantitative Medical Imaging Section National Institute of Biomedical Imaging and Bioengineering National Institutes of Health Bethesda MD USA; ^2^ Henry M. Jackson Foundation for the Advancement of Military Medicine, Inc. Bethesda MD USA; ^3^ Center for Neuroscience and Regenerative Medicine, Uniformed Services University Bethesda MD USA; ^4^ Laboratory of Molecular Signaling National Institute on Alcohol Abuse and Alcoholism National Institutes of Health Bethesda MD USA; ^5^ Georgia Cancer Center Augusta University Augusta GA USA; ^6^ Department of Radiology and Radiological Sciences Uniformed Services University Bethesda MD USA

**Keywords:** closed‐head injury, diffusion tensor imaging, mouse, MRI, omega‐3 fatty acids, traumatic brain injury

## Abstract

Previous studies suggest that long‐term supplementation and dietary intake of omega‐3 polyunsaturated fatty acids (PUFAs) may have neuroprotective effects following brain injury. The objective of this study was to investigate potential neuroprotective effects of omega‐3 PUFAs on white matter following closed‐head trauma. The closed‐head injury model of engineered rotational acceleration (CHIMERA) produces a reproducible injury in the optic tract and brachium of the superior colliculus in mice. Damage is detectable using diffusion tensor imaging (DTI) metrics, particularly fractional anisotropy (FA), with sensitivity comparable to histology. We acquired *in vivo* (*n* = 38) and *ex vivo* (*n* = 41) DTI data in mice divided into sham and CHIMERA groups with two dietary groups: one deficient in omega‐3 PUFAs and one adequate in omega‐3 PUFAs. We examined injury effects (reduction in FA) and neuroprotection (FA reduction modulated by diet) in the optic tract and brachium. We verified that diet did not affect FA in sham animals. In injured animals, we found significantly reduced FA in the optic tract and brachium (~10% reduction, *p* < 0.001), and Bayes factor analysis showed strong evidence to reject the null hypothesis. However, Bayes factor analysis showed substantial evidence to accept the null hypothesis of no diet‐related FA differences in injured animals in the *in vivo* and *ex vivo* samples. Our results indicate no neuroprotective effect from adequate dietary omega‐3 PUFA intake on white matter damage following traumatic brain injury. Since damage from CHIMERA mainly affects white matter, our results do not necessarily contradict previous findings showing omega‐3 PUFA‐mediated neuroprotection in gray matter.

## Graphical abstract

We investigated potential neuroprotective effects of dietary omega‐3 polyunsaturated fatty acids (PUFAs) on white matter damage following closed‐head injury in mice. While injured animals exhibited reduced fractional anisotropy (FA) in the optic tract and brachium compared to the uninjured animals, there was no evidence of neuroprotection from dietary omega‐3 PUFAs.

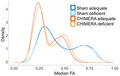

**Significance**

Omega‐3 fatty acid supplementation has shown positive, anti‐inflammatory effects following stroke and traumatic brain injury (TBI). Positive effects on these high incidence and prevalence conditions have contributed to the popularity of omega‐3 fatty acids with a $13 billion global market. Recent research on heart disease and cancer, however, suggest that omega‐3 fatty acids may not provide the health benefits that were initially expected. In the brain, there is still an exclusive predominance of positive results. To our knowledge, our study is the first that casts doubt that omega‐3 fatty acid supplementation may have neuroprotective effects on tissue damage from TBI.

## 1 INTRODUCTION

Polyunsaturated fatty acids (PUFA)s are important in the brain's inflammatory response (Bazinet & Layé, 2014; Layé, 2010; Layé, Nadjar, Joffre, & Bazinet, 2017; Trépanier, Hopperton, Orr, & Bazinet, 2016). Omega‐6 and omega‐3 PUFAs compete for incorporation into cell membranes and are both released following membrane damage (Surette, 2008). The relative concentration of omega‐6 and omega‐3 PUFAs is important for inflammation since once they are released from the membrane, omega‐6 PUFAs have pro‐inflammatory effects, while omega‐3 PUFAs have anti‐inflammatory effects (Bazán, 1970; Rehncrona, Westerberg, Åkesson, & Siesjö, 1982; Yoshida et al., 1986). The ratio of omega‐6 to omega‐3 PUFAs in the human diet can be highly imbalanced (~20 to 1; Simopoulos, 2016), resulting in higher levels of omega‐6 PUFAs relative to omega‐3 PUFAs in cell membranes that can exacerbate the inflammatory response.

In rodent injury models, long‐term omega‐3 PUFA supplementation reduces omega‐6 PUFA metabolites released from damaged tissue and offers anti‐inflammatory effects (Cao et al., 2006, 2007). These effects include decreased pro‐inflammatory microglial cell density and cytokine activity in damaged tissue. In ischemia and traumatic brain injury (TBI), these anti‐inflammatory effects reduce neuronal damage and cell death that preserve gray matter and improve cognitive and behavioral outcomes (Cao et al., 2007; Desai et al., 2016; Desai, Kevala, & Kim, 2014; Fernandes, Mori, Ekuni, Oliveira, & Milani, 2008; Lalancette‐Hébert et al., 2011; Okada et al., 1996; Pan et al., 2009; Zhang et al., 2014; Zhang, Hu, Yang, Gao, & Chen, 2010).

While several studies suggest that omega‐3 PUFAs could be protective in gray matter, evidence of neuroprotection in white matter is lacking. The investigation of potential neuroprotective effects of omega‐3 PUFA in white matter is important for human TBI, since human TBI often includes white matter damage known as diffuse axonal injury (DAI; Adams et al., 1989; Johnson, Stewart, & Smith, 2013). Few results from rodent TBI models suggest that omega‐3 PUFAs may reduce axonal damage by different mechanisms, including lowering the levels of beta amyloid precursor protein and nonphosphorylated neurofilament protein (Bailes & Mills, 2010; Mills, Bailes, Sedney, Hutchins, & Sears, 2010; Mills, Hadley, & Bailes, 2011), preserving myelin by maintaining levels of myelin basic protein, and protecting oligodendrocytes from excitotoxicity damage (Pu et al., 2013, 2017). However, these effects have been observed in the Marmarou and controlled cortical impact (CCI) injury models that do not biomechanically resemble human TBI and do not produce comparable white matter damage (Namjoshi et al., 2013), making it difficult to extend protective effects of omega‐3 PUFAs to human injury.

The closed‐head injury model of engineered rotational acceleration (CHIMERA) was developed as a reproducible model that mimics the white matter damage observed in human TBI (Namjoshi et al., 2014). Histological assessment identified the optic tract and brachium of the superior colliculus as two regions severely affected by CHIMERA. Silver‐staining revealed prominent axonal injury and glial fibrilary acidic protein (GFAP) and ionized calcium‐binding adapter molecule 1 (Iba‐1) immunohistochemistry indicated increased numbers of glia in these regions (Haber et al., 2017; Namjoshi et al., 2014). *Ex vivo* diffusion tensor imaging (DTI) showed that fractional anisotropy (FA) was significantly reduced in the optic tract and brachium of the superior colliculus in the injured animals that corresponded with axonal degeneration and gliosis observed in the histological analysis (Haber et al., 2017). This previous study found that diffusivity measures (Trace, AD, RD) were not as sensitive as FA to the damage, and validated the use of FA as a biomarker with specificity and sensitivity comparable to that of histology to identify damage from CHIMERA.

The main objective of this study is to investigate a potential neuroprotective effect of a diet adequate in omega‐3 PUFAs on white matter damage from closed‐head TBI. CHIMERA is an ideal model because it is reproducible, damage is specifically in white matter, and we have data on the specificity and sensitivity of FA as a biomarker for damage from CHIMERA in the optic tract and brachium of the superior colliculus. In this study, we examine FA values from *ex vivo* DTI to replicate injury effects observed previously (Haber et al., 2017), then investigate neuroprotective effects of the adequate diet. Because our ultimate goal is to apply these techniques to studying human TBI, we performed an identical analysis *in vivo* to determine if effects observed *ex vivo* can be detected noninvasively *in vivo*.

## 2 MATERIALS AND METHODS

### 2.1 Experimental subjects

The study included adult C57BL/6 male mice (Charles River Laboratories, Wilmington, MA) that were fed either a diet with adequate omega‐3 PUFA levels or deficient omega‐3 PUFA levels and received either repeated CHIMERA or sham injury. The diets were manipulated such that the omega‐6 PUFA composition was similar in the adequate and deficient diets (linoleic acid = 18.2% in the deficient diet vs. 17.2% in the adequate diet) and differed substantially in omega‐3 PUFA composition (alpha‐linolenic acid = 0.04% in the deficient diet vs. 3.8% in the adequate diet). Additional nonfatty acid ingredients were identical between the two diets. The omega‐3 PUFA‐deficient diet began during gestation resulted in a 35% depletion of omega‐3 PUFAs (Desai et al., 2016). These diets were maintained following CHIMERA for the duration of the study period. The normal 12‐hr light period was maintained in the animal housing facility, and mice had free access to food and water.

The *in vivo* sample included 38 mice with sham‐adequate *n* = 9, sham deficient *n* = 12, CHIMERA‐adequate *n* = 9, CHIMERA deficient *n* = 8. The *ex vivo* sample included 41 mice with sham‐adequate *n* = 9, sham deficient *n* = 14, CHIMERA‐adequate *n* = 11, CHIMERA deficient *n* = 7. Thirty‐three animals were in common between the *ex vivo* sample and the *in vivo* sample.

#### 2.1.1 Sample size estimation

CHIMERA produces a highly reproducible injury with tissue damage that is observable by eye with evident silver staining in the optic tract and brachium of the superior colliculus at the individual level (Haber et al., 2017; Namjoshi et al., 2014). A previous *ex vivo* DTI study (Haber et al., 2017) showed a measurable and significant effect of CHIMERA in these same regions in a relatively small sample (injured animal = 5, sham animal = 5). Based on the results of this previous *ex vivo* DTI experiment, we set our minimum sample size at *N* = 5 and added additional animals to account for the possible introduction of variability from noise or other factors in the *in vivo* scans as well as to account for possible animal attrition. We did not perform a formal power analysis a priori since this would require a full characterization of experimental variability and population variability, which was not possible in this case since the previous *ex vivo* DTI study was performed on a different scanner with a different DTI acquisition scheme.

### 2.2 Ethical approval

Experiments in this study were approved by the National Institute on Alcohol Abuse and Alcoholism Animal Care and Use Committee (ACUC) and the Uniformed Services University of the Health Sciences Institutional ACUC, and were conducted in accordance with the National Institute of Health Guide for the Care and Use of Laboratory Animals and the National Research Council Guide to the Care and Use of Laboratory Animals.

### 2.3 Experimental design and procedures

#### 2.3.1 CHIMERA procedure

The mice underwent repeated CHIMERA or sham injury at 3–4 months old (Namjoshi et al., 2014). Prior to the procedure, the mice were anesthetized with 5% isoflurane for 2 min in oxygen (1L/min) and positioned supine on the holding platform of the CHIMERA apparatus angled at ~32 degrees to place the top of the head flat over a hole in the head plate. The mouse head was aligned so that the impact was limited to the dorsal cortical region in the 5 mm area surrounding bregma. The input kinetic energy (Ek) was calculated using the formula Ek = 1/2 mv^2^ (v = velocity (m/sec), m = mass of piston). In this study, a 0.55J kinetic energy was delivered to the mouse head using a 50‐g piston. Anesthesia was discontinued once the impact occurred. The injured mice received three impacts 24 hr apart. The sham mice were given all the procedures except for the impact. After each impact, the mice were monitored and were continuously observed until recovery. The injured mice did not exhibit fractures, hematomas, apnea, or death following the impact.

#### 2.3.2 Sample preparation for *ex vivo* imaging

Following the *in vivo* MRI acquisition, each mouse was administered an i.p. overdose of Euthasol (50 mg/kg). Once the mouse no longer responded to a toe pinch, it was transcardially perfused with 250 ml of ice‐cold phosphate‐buffered saline (PBS, pH 7.4) containing 2% heparin (Sigma‐Aldrich) followed by 250 ml of ice‐cold 4% paraformaldehyde solution in PBS (Santa Cruz Biotechnology). The skull was then removed from the body and was postfixed in the 4% paraformaldehyde solution for 24 hr and then transferred to a solution containing 0.01% sodium azide in PBS for storage. After at least 1 week of rehydration in this solution, the soft tissue and mandibles were removed from the skulls, and the remaining cranium was immersed in Fluorinert (FC‐3283, 3M, St. Paul, MN, United States) in a 5‐ml plastic syringe for imaging.

#### 2.3.3 Image acquisition and processing

Imaging was performed on the mice in the chronic period (greater than 3 months) following injury with repeated CHIMERA or sham injury. Both *in vivo* and *ex vivo* imaging acquisitions were performed on a 7T small animal (20‐cm horizontal bore) Bruker BioSpec scanner (Billerica, MA, USA) with 120‐mm diameter, 670 mT/m gradient coils using the ParaVision 6.0.1 software.

The *in vivo* imaging was performed with a 20‐mm quadrature volume coil (RAPID Biomedical, Rimpar, Germany) for transmit and receive. For *in vivo* diffusion MRI, single shot 2‐D echo planar imaging (2D‐EPI) with echo time (TE)/repetition time (TR) = 50–60/5,000–6,000 ms was used for a coronal acquisition of 84 volumes with FOV = 14.4 mm × 19.2 mm and matrix = 72 × 96 to yield 0.2 mm × 0.2 mm in‐plane resolution with 24 slices of 0.4 mm slice thickness. The diffusion sampling scheme included one shell with four *b* = 0 images and *b* = 100, 200, and 1,100 s/mm^2^ with 12 directions (6 directions plus 6 directions antipodal to them), and another shell with four *b* = 0 images and *b* = 1,100 s/mm^2^ with 40 directions (20 directions plus 20 antipodal to them). To correct for EPI distortions, two repetitions for each diffusion‐weighted image (DWI) were collected with opposite phase‐encode directions for use with diffeomorphic registration for blip‐up blip‐down diffusion imaging (DR‐BUDDI) processing (Irfanoglu et al., 2015).

The *ex vivo* imaging was performed with a 15‐mm solenoid coil for transmit and receive. For *ex vivo* diffusion MRI acquisition consisted of multishot 3‐D echo planar imaging (3D‐EPI) with eight segments and TE/TR = 30/900 ms, FOV = 12.6 × 17.5 × 8.4 mm and matrix = 84 × 117 × 56 resulting in isotropic voxel dimensions of 0.15 × 0.15 × 0.15 mm. DWI sampling consisted of *b*(s/mm^2^)/gradient direction number: 500/6, 1,500/24, 3,000/24, 4,500/48 with three *b* = 0 images that yielded 105 volumes. An additional *b* = 500 shell with one *b* = 0 image was collected with opposite phase‐encode direction for use with DR‐BUDDI processing (Irfanoglu et al., 2015).

T2 RARE images were also acquired for both *in vivo* and *ex vivo* for use in the DR‐BUDDI correction. The *in vivo* T2 acquisition performed with 2‐D multiecho RARE imaging was used with TE values of 10, 30, 50, 70, 90, 110 ms, and TR = 4,000. A coronal acquisition was used with FOV = 14.4 mm × 19.2 mm, matrix = 72 × 96, 24 slices with slice thickness = 0.4 mm, and number of experiments (NEX) = 1. The *ex vivo* T2 acquisition was acquired at the same special geometry and dimensions as the *ex vivo* DWIs, with TE = 10, 30, 50, 70, 90 ms, and TR = 4,000. TE = 30 was used for both *ex vivo* and *in vivo* DR‐BUDDI correction. The total *in vivo* scan time for each animal was 1 hr and 45 min, while the total *ex vivo* scan time was 15 hr and 11 min.

The raw *in vivo* DWIs were imported and preprocessed with the TORTOISE DTI pipeline v3.1.4, while raw *ex vivo* DWIs were processed with the TORTOISE DTI pipeline v3.2.0. Both versions of the pipeline included motion and eddy distortion correction and DR‐BUDDI correction for EPI distortions (Irfanoglu et al., 2015; Irfanoglu, Nayak, Jenkins, & Pierpaoli, 2017; Pierpaoli et al., 2010). For the *in vivo* and *ex vivo* data, the diffusion tensor was fit using the DIFFCALC v2.5 module of TORTOISE and was used to generate FA (Basser & Pierpaoli, 1996) and directionally encoded color (DEC) maps (Pajevic & Pierpaoli, 1999).

Separate study‐specific DTI templates were created for *in vivo* and *ex vivo* images using diffeomorphic registration for tensor accurate alignment of anatomical structures (DR‐TAMAS), and the resulting deformation fields were used to put the native scalar maps into the study‐specific DTI template space (Irfanoglu et al., 2016).

#### 2.3.4 Optic tract and brachium region of interest (ROI)

A specific ROI encompassing the optic tract and brachium of the superior colliculus was used for this analysis. This ROI was defined because these two regions had been shown to be the most severely injured in CHIMERA in a previous *ex vivo* study (Haber et al., 2017). This ROI was manually drawn on each study‐specific template (*in vivo* and *ex vivo* templates) using anatomical guidance about the tract location from DEC maps and the Allen mouse brain atlas (Lein et al., 2007).

#### 2.3.5 Statistical analysis

The central focus of this study was to investigate a potential neuroprotective effect of a diet adequate in omega‐3 PUFAs on white matter damage after a closed‐head brain injury. As a measure of white matter damage, we computed median FA value in the optic tract and brachium ROI for each animal in both the *ex vivo* and *in vivo* samples.

We included data from all acquired scans in our analyses. The analyses were performed in R using ANTsR (v0.7.1) for reading and writing images, Tidyverse (v1.2.1) packages for organizing and plotting data, and base R statistics (v3.4.2) and BayesFactor (0.9.12‐4.2) for statistical analyses (Avants, 2017; Morey & Rouder, 2018; R Core Team, 2017; Wickham, 2017). Identical analyses were performed in the *ex vivo* and *in vivo* samples. For one‐tailed tests, we used *α* = 0.025, while for two‐tailed tests *α* = 0.05. We performed a multiple comparisons correction for all *p* values obtained in the study using a false discovery rate (FDR) correction.

**FIGURE 1:**
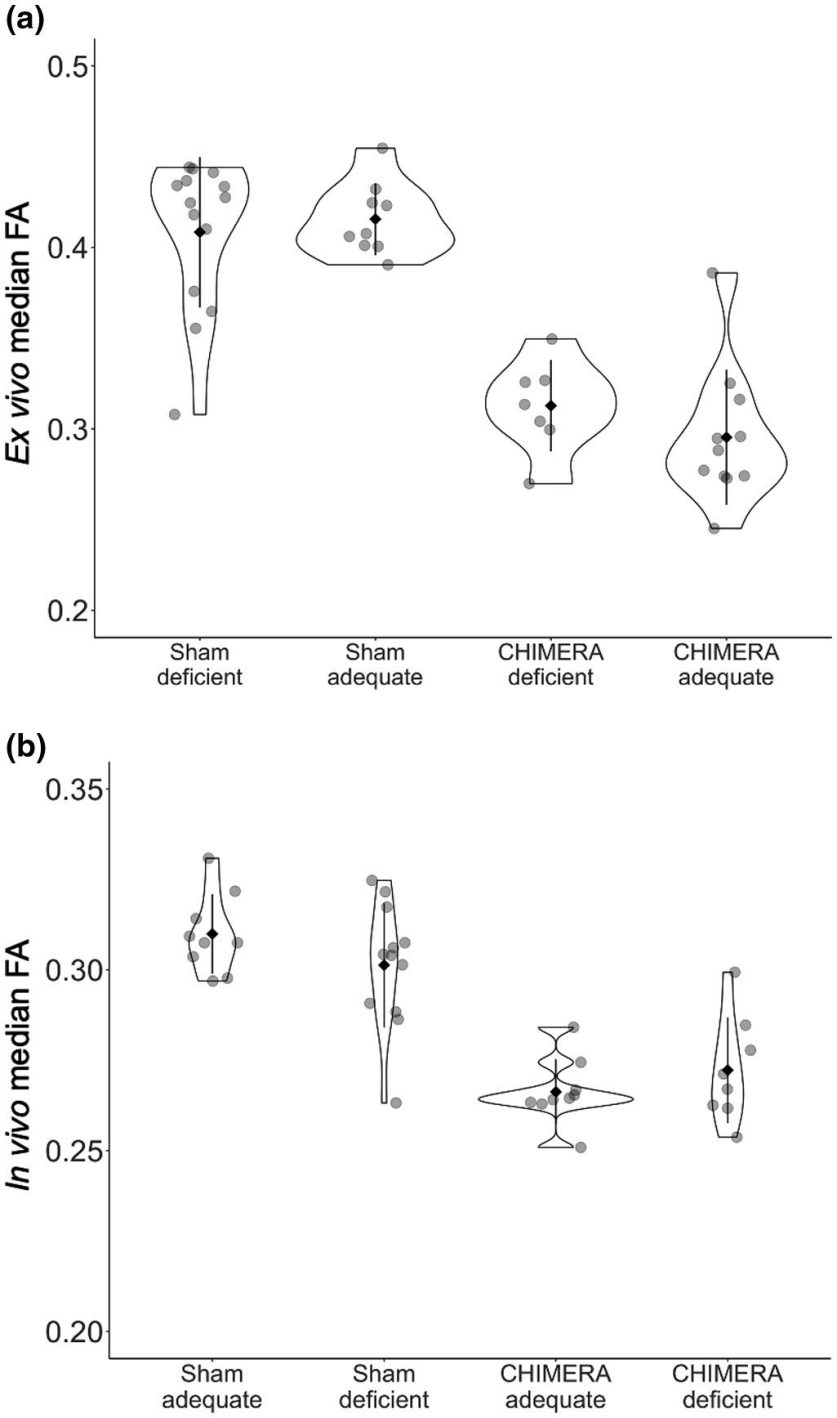
Violin plots showing the distributions of the median FA values in the optic tract and brachium ROI by diet and injury group for *ex vivo* (a) and *in vivo* (b), with the mean (solid diamond) ± standard deviation (bars), and individual data points (transparent dots). For the *ex vivo* sample, we did not detect a significant diet effect in the sham group, and the Bayes factor indicated anecdotal evidence to accept the null hypothesis (BF = 0.419). Both diet groups showed significantly lower FA in the CHIMERA group compared to the sham group, and the Bayes factors showed strong evidence to reject the null hypothesis (deficient BF = 48.7 × 10^4^, adequate BF = 79.8). The CHIMERA deficient and CHIMERA‐adequate groups were not significantly different and the Bayes factor indicated substantial evidence to accept the null hypothesis (BF = 0.241). There was large overlap between the two groups. The *in vivo* sample showed similar results as the *ex vivo* sample with no significant diet effect (BF = 0.715), significant injury effects in both diet groups (deficient BF = 1.90 × 10^5^, adequate BF = 59.0), and no significant difference between the CHIMERA deficient and CHIMERA‐adequate groups (BF = 0.247), indicating no neuroprotective effect of diet

To assess a potential neuroprotective effect of omega‐3 PUFAs, we first needed to rule out the possibility that the adequate diet itself may affect FA values. Since diet had never been investigated on its own and we had no prior evidence for how diet may affect FA values, this component of the study was exploratory. We performed our analysis in the sham animals. To examine the diet effect, we used a two‐tailed Welch *t*‐test to investigate differences in FA between the adequate and deficient diets in the sham group, that is, differences in group means (adequate vs. deficient) of the distributions of median FA measured for the individual animals (see Figure 1). A statistically significant result would indicate a non‐negligible diet effect that would need to be accounted for when interpreting the results from the subsequent analyses. We also computed Hedge's *g* (difference of group means/pooled standard deviation weighted by sample size) using adequate sham‐deficient sham for the numerator (Hedges, 1981).

Next, we needed to confirm that we are able to detect the injury effect and replicate the findings from Haber et al. (2017). Based on this previous work, we expect that the median FA values will be lower in injured animals compared to the sham animals. To test this hypothesis, we performed one‐tailed Welch *t*‐tests separately within the adequate and deficient diet groups to test if median FA values in the injured group were lower than those in the sham group, and computed Hedge's *g* for each comparison (CHIMERA—sham/pooled standard deviation weighted by sample size). We performed the tests separately in each diet group assessing differences in group means (injured vs. sham) of median FA of the individual animals to take into account any potential neuroprotective effect of the diet that might affect the severity of the injury.

We focused on the injured animals to examine the potential neuroprotective effect of omega‐3 PUFAs. We formed our hypothesis to test for a neuroprotective effect of the adequate diet based on the previous results from Haber et al. (2017) that showed that the injury resulted in reduced FA. If the adequate diet offers the white matter neuroprotection against damage, the injured animals on the adequate diet should have FA values that are more similar to those of the unaffected sham animals. Therefore, we expect that the injured animals on the adequate diet should have higher median FA than the injured animals on the deficient diet. We applied a one‐tailed Welch *t*‐test to test if the CHIMERA‐adequate group had higher median FA than the CHIMERA‐deficient group, based on the group means of median FA of the individual animals of those groups. Significantly higher median FA in the CHIMERA‐adequate group would indicate a neuroprotective effect of the diet on white matter damage resulting from CHIMERA. We also computed Hedge's *g* for this comparison (adequate CHIMERA—deficient CHIMERA/pooled standard deviation weighted by sample size).

The previous tests we performed fall within the framework of null hypothesis significance testing (NHST). In NHST, a null hypothesis, or no effect, is proposed and the data are then tested to assess if it conforms to the null hypothesis at a given significance level, or *p* value (Pernet, 2016). The *p* value, however, only expresses the probability of obtaining a result as extreme as the one observed assuming that the null hypothesis is true, and does not express the magnitude of the effect nor the strength of evidence for accepting or rejecting the null hypothesis (Krzywinski & Altman, 2013).

To assess the level of confidence we have in accepting or rejecting the null hypothesis, we can compute Bayesian inference to examine the weight of evidence for the null hypothesis compared to an alternate hypothesis of a nonzero effect (Faulkenberry, 2018). Bayes' theorem shows the probability of a hypothesis (*H*) given the data that were collected (*D*), or posterior probability, as a ratio of the likelihood of the data (*D*) given the hypothesis (*H*) multiplied by the prior probability of the hypothesis (*H*), and the probability of the collecting the data (*D*):

$$p(H|D)=\frac{p(D|H)\cdot p\left(H\right)}{p\left(D\right)}.$$

To compare the relative probability of two different hypothesis, such as the null hypothesis (*H_0_*) for no effect and an alternative hypothesis (*H_A_*) for a nonzero effect, Bayes' theorem can be applied in the following manner:

$$\frac{p(H_{0}\left|D\right)}{p(H_{A}\left|D\right)}=\frac{p(D|H_{0})}{p(D|H_{A})}\cdot \frac{p\left(H_{0}\right)}{p\left(H_{A}\right)}.$$

For our study, we assume the same prior probability (1:1) for both hypotheses since we believe that the null and alternative hypotheses are equally likely. Therefore, any change in the posterior probability ratio compared to the prior probability (i.e., any deviation from 1:1) would result from a change in the likelihood of one hypothesis compared to the other. This change in the ratio of likelihoods is the Bayes factor, and it provides a means of assessing the weight of evidence for either hypothesis (Kass & Raftery, 1995; Jeffreys, 1961). A chart with possible Bayes factor values and their interpretations is shown in Table 1. In addition to each of the NHST Welch *t*‐tests described previously, we also employed Bayes factor analyses on the same contrasts to assess the weight of evidence for the null and alternate hypotheses. Since the test to examine diet effect was two‐tailed, the corresponding Bayes factor analysis used a two‐sided distribution to determine the Bayes factor. All other tests were one‐tailed and used the positive portion of the distribution to test for positive effect size of the sham compared to the CHIMERA groups.

**TABLE 1:** Bayes factors and interpretations^a^

Bayes factor	Interpretation
>100	Decisive evidence for *H_A_*
30–100	Very strong evidence for *H_A_*
10–30	Strong evidence for *H_A_*
3–10	Substantial evidence for *H_A_*
1–3	Anecdotal evidence for *H_A_*
1	No evidence
1/3–1	Anecdotal evidence for *H*_0_
1/10–1/3	Substantial evidence for *H*_0_
1/3–1/10	Strong evidence for *H*_0_
1/100–1/30	Very strong evidence for *H*_0_
<1/100	Decisive evidence for *H*_0_

^a^ Table from Faulkenberry (2018), adapted from Jeffreys (1961).

The distribution of FA values in an ROI in brain parenchyma is typically positively skewed. Higher FA values in the tail of the distribution come from voxels less contaminated by CSF and gray matter partial volume and therefore are expected to be more representative of the effects of diet or injury in white matter. In our previous analyses, we performed tests using the median FA value within the optic tract and brachium ROI from each animal. While these median values are capable of showing the central tendency of the FA values in the ROI, they may not properly describe potential differences in the tails of the distribution of FA values in the ROI.

Therefore, to better examine the diet, injury, and potential neuroprotective effects of the diet in the optic tract and brachium ROI, we compared the distributions of FA values as a function of diet and injury by group using Kolmogorov–Smirnov (K–S) tests. We were specifically interested in examining how the overall voxel‐wise distribution of FA values differed across the ROI between the diet groups, rather than a single summary value for the ROI as we did with the previous set of parametric tests. Given that the distribution of FA values is skewed within the ROI, the K–S tests were performed to account for the remote possibility that white matter damage would have affected preferentially the highest FA values (tail of the distribution within the ROI) and not the median of the distribution. For this test, we computed the median values for each voxel in the ROI for each group (i.e., sham deficient, sham‐adequate, CHIMERA deficient, CHIMERA‐adequate). We performed a two‐sided K–S test on the voxel‐wise group median FA values for the diet effect in the sham group, and one‐sided K–S tests on the voxel‐wise group median FA values for injury effects in the adequate and deficient diet groups (CHIMERA‐adequate—sham‐adequate, CHIMERA deficient—sham deficient), and neuroprotective effect in the injured group (CHIMERA‐adequate—CHIMERA deficient).

In addition to the ROI analysis, we computed voxel‐wise effect size maps to visually inspect the parenchyma for any clusters or patterns in the brachium and optic tract region that would indicate any effects of diet or injury. Using a voxel‐wise map allowed for a comprehensive representation of the diet and injury effects that allowed us to survey the parenchyma without bias or observer‐dependent intervention in selecting an ROI. We created the effect size maps with fslmaths from FSL software (Jenkinson, Beckmann, Behrens, Woolrich, & Smith, 2012; Smith et al., 2004). We computed Hedges' *g* for each metric, as described previously. For diet, the maps were computed as (adequate diet—deficient diet)/pooled standard deviation weighted by sample size. We computed the effect size maps for the injury effect in a similar manner: (CHIMERA—sham)/pooled standard deviation weighted by sample size.

## 3 RESULTS

### 3.1 Diet effect

**FIGURE 2:**
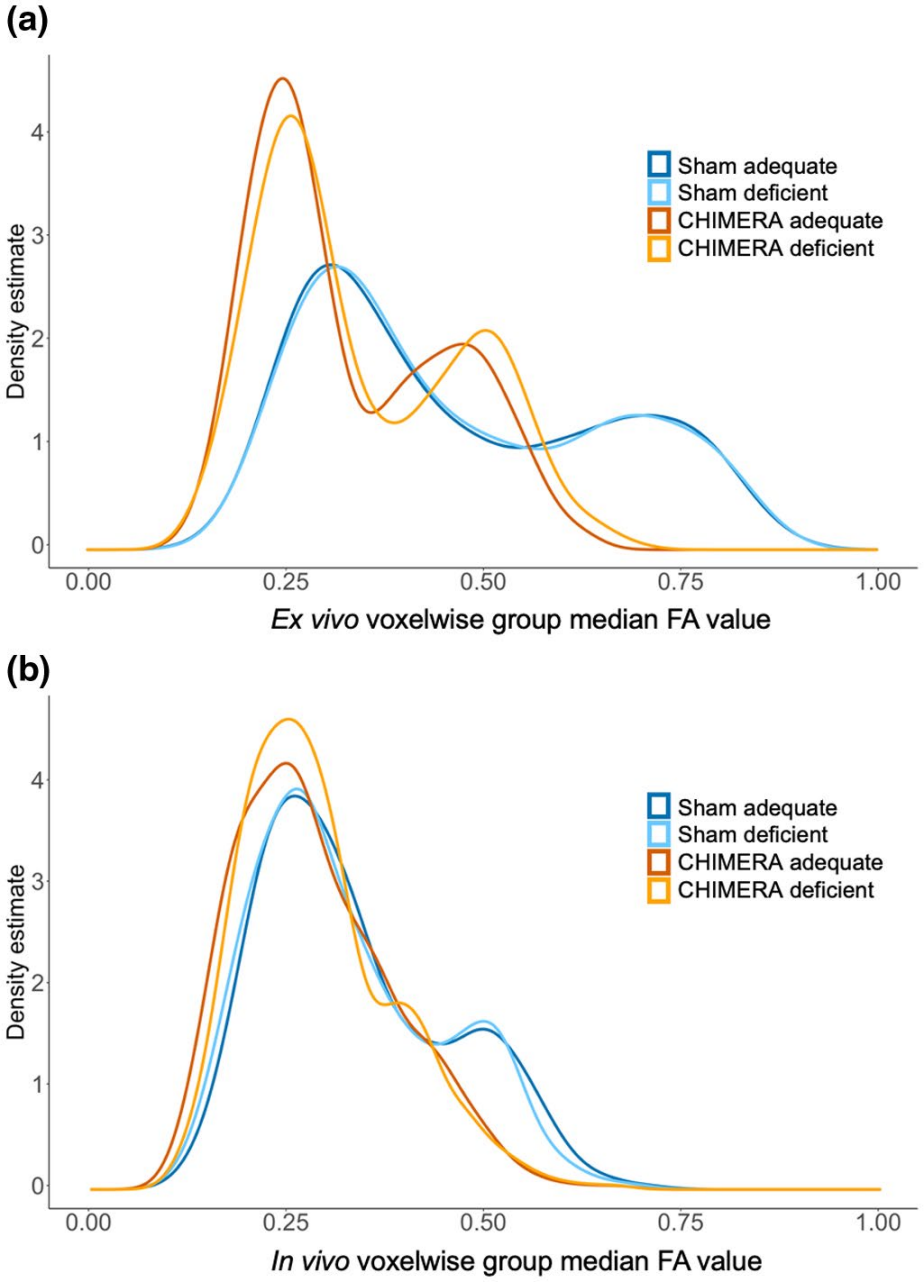
Density plots of voxel‐wise group median FA values by diet and injury group for *ex vivo* (a) and *in vivo* (b) samples in the optic tract and brachium ROI. For the *ex vivo* sample, the distributions of voxel‐wise median FA values in the sham‐adequate and sham‐deficient groups overlapped and were nearly identical, indicating no effect of diet. Both sham groups had positively shifted distributions compared to their corresponding CHIMERA groups, showing a strong injury effect. The *in vivo* sample showed similar results, though the CHIMERA‐adequate and CHIMERA deficient distributions showed greater similarity

**FIGURE 3:**
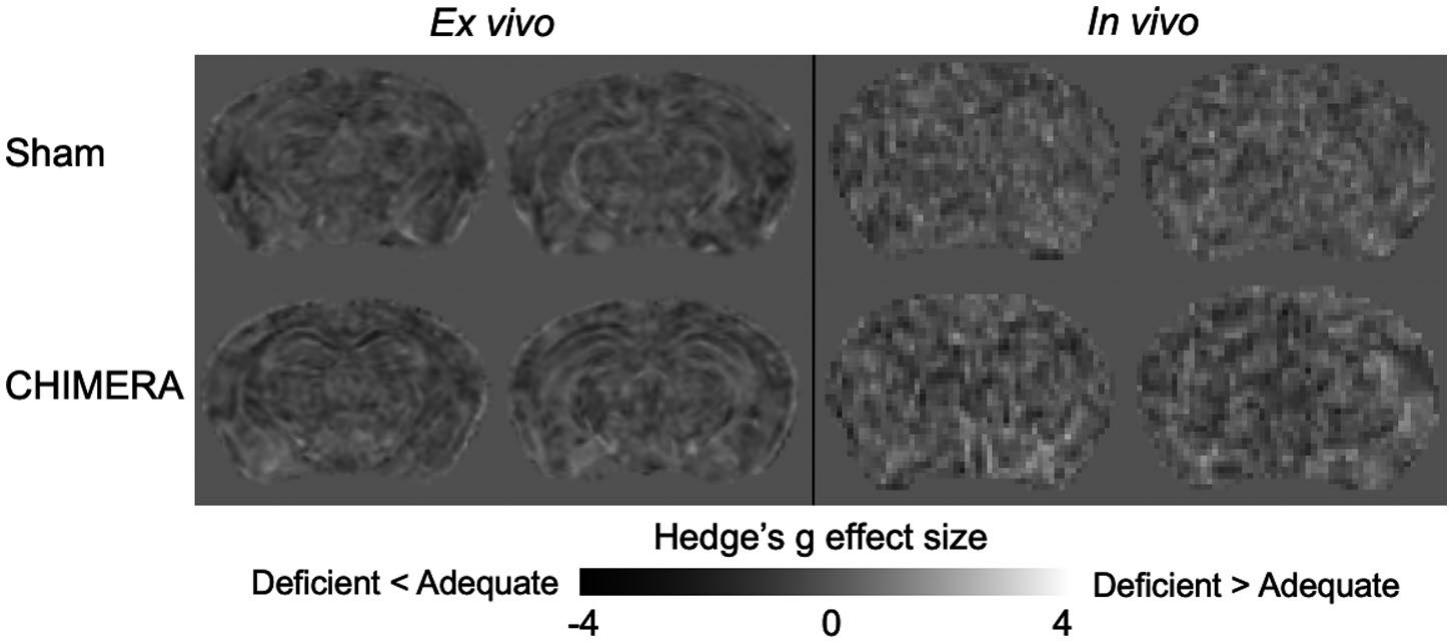
Diet effect size maps showing voxel‐wise Hedges' *g* in the sham group for *ex vivo* and *in vivo* samples. Maps depict the (adequate diet group—deficient diet group)/pooled standard deviation, with positive values (lighter gray) showing where the adequate group had higher FA than the deficient group, and negative values (darker gray) showing where the adequate group had lower FA than the deficient group. There are no clusters or patterns indicating diet effects in the optic tract or brachium of the superior colliculus in either the *ex vivo* and *in vivo* effect size maps, and both maps appear relatively flat

We found no detectable effect of diet in the optic tract and brachium ROI. In the *ex vivo* sample, FA values in the sham‐adequate group were not significantly higher than those in the sham‐deficient group and showed a negligible effect size (Table 2, Figure 1a), while the Bayes factor for this comparison suggested anecdotal evidence for accepting the null hypothesis. A comparison of the distributions using a K–S test indicated that there was no significant difference between voxel‐wise median FA values in the ROI (Table 3), and the distributions are identical in shape and overlap almost entirely (Figure 2a). The effect size maps for the diet effect did not show any voxels in the optic tract and brachium that were affected by diet in the sham group, CHIMERA group, or the whole combined group (Figure 3). Based on these results, we do not have strong evidence that diet itself independently modulates FA.

**TABLE 2:** Two‐tailed Welch *t*‐test results for sham‐adequate FA versus sham deficient FA

	Group	*Ex vivo*	*In vivo*
*N*	Sham deficient	14	12
	Sham‐adequate	9	9
Mean	Sham deficient	0.408	0.301
	Sham‐adequate	0.416	0.310
*t* value		0.560	1.40
Degrees of freedom		19.8	18.6
FDR‐adjusted *p* value		1.00	1.00
Hedge's *g* effect size (Adequate—Deficient)		0.200	0.555
Bayes factor		0.419	0.715

**TABLE 3:** Kolmogorov–Smirnov test results

Scan type	Contrast	Group 1 N	Group 2 N	D^a^	FDR‐adjusted *p* value
*Ex vivo*	SHAM‐adequate vs. SHAM deficient^b^	9	14	0.012	1.00
	CHIMERA‐adequate vs. SHAM‐adequate	11	9	0.312	<0.001
	CHIMERA deficient vs. SHAM deficient	7	14	0.282	<0.001
	CHIMERA‐adequate vs. CHIMERA deficient	11	7	0.0005	1.00
*In vivo*	SHAM‐adequate vs. SHAM deficient^b^	9	12	0.054	1.00
	CHIMERA‐adequate vs. SHAM‐adequate	9	9	0.168	<0.001
	CHIMERA deficient vs SHAM deficient	8	12	0.147	<0.001
	CHIMERA‐adequate vs CHIMERA deficient	9	8	0.035	1.00

^a^ D = Kolmogorov–Smirnov distance statistic.

^b^ Two‐sided comparison.

Similar to the *ex vivo* results, the *in vivo* results did not show a significant effect of the diet on FA with only a medium effect (Table 2, Figures 1b and 3), and the Bayes factor showed anecdotal evidence to accept the null hypothesis (Table 2). The K–S test also did not show a significant difference between the diets (Table 3), and the distributions of the sham‐adequate and sham‐deficient groups were similar (Figure 2b).

### 3.2 Injury effect

**FIGURE 4:**
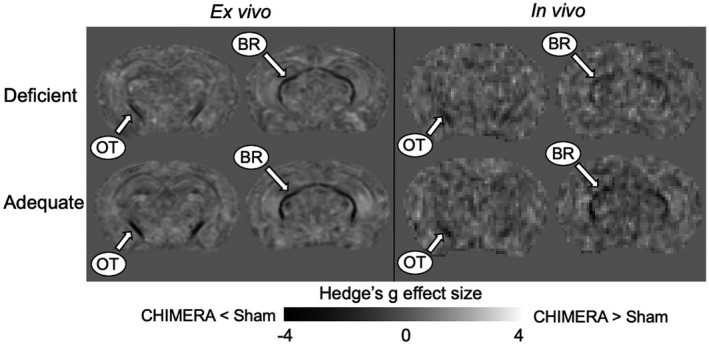
Injury effect size maps showing voxel‐wise Hedges' *g* for the *ex vivo* and *in vivo* samples. The effect size maps were calculated by (CHIMERA group—sham group)/pooled standard deviation, with positive values (lighter gray) showing where the CHIMERA group had higher FA than the sham group, and negative values (darker gray) showing where the CHIMERA group had lower FA than the sham group. Both the *ex vivo* and *in vivo* effect size maps show a pattern of dark voxels indicating negative values throughout the optic tract and brachium of the superior colliculus (BR), replicating previous results found in FA from Haber et al. (2017)

We replicated the previous results from Haber et al. (2017) in the *ex vivo* sample and detected a strong injury effect (low FA) in the optic tract and brachium of the superior colliculus. FA values in the injured group were significantly lower than those of the sham group for both diet groups with the effect size showing a large diet effect (Table 4, Figure 1a). Bayes factors for the adequate comparison showed decisive evidence for rejecting the null hypothesis, while the deficient comparison showed very strong evidence. A K–S test also indicated significantly higher FA in the sham group compared to the CHIMERA group in both diets (Table 3), and a plot showed that the distributions of sham and CHIMERA voxel‐wise median group FA values in the ROI did not overlap for either diet (Figure 2a). The injury effect size maps show a robust FA decrease in the optic tract and brachium for the deficient and adequate diet groups (Figure 4).

**TABLE 4:** One‐tailed Welch *t*‐test results for sham > CHIMERA in each diet group

	Group	*Ex vivo*		*In vivo*	
		Deficient	Adequate	Deficient	Adequate
*N*	Sham	14	9	12	9
	CHIMERA	7	11	8	9
Mean	Sham	0.409	0.415	0.301	0.310
	CHIMERA	0.313	0.295	0.272	0.266
*t* value		6.55	9.22	4.05	9.21
Degrees of freedom		18.1	15.8	16.8	15.4
FDR‐adjusted *p* value		<0.001	<0.001	<0.001	<0.001
Hedge's *g* effect size (CHIMERA—Sham)		2.48	3.74	1.71	4.14
Bayes factor		1.36 × 10^4^	2.26 × 10^5^	59.0	1.90 × 10^5^

The injury effect from Haber et al. (2017) was also replicated in the *in vivo* data with significantly higher FA values in the sham group compared to the injured group with a large effect size (Table 3, Figures 1b and 3), and the Bayes factors indicated strong evidence to reject the null hypothesis (Table 4). The K–S also detected a significant injury effect in both diet groups (Table 3), with the distributions showing nonoverlapping distributions between the sham and CHIMERA groups (Figure 2b).

### 3.3 Neuroprotective effect

We did not detect a neuroprotective effect of the adequate diet following the injury in the *ex vivo* sample. There was no significant difference in FA between the adequate and deficient CHIMERA groups with the effect size showing a medium effect in the opposite direction that we hypothesized in our one‐sided *t*‐test (deficient CHIMERA FA > adequate CHIMERA FA), and the Bayes factor showing substantial evidence to accept the null hypothesis. The K–S test also showed no difference between the two groups (Table 3), and the distributions of the two groups were largely overlapping (Figure 2a).

We did not detect a neuroprotective effect in the *in vivo* sample. The *in vivo* data also showed no significant difference in FA between the CHIMERA‐adequate and deficient groups with a medium effect size in the opposite direction of the *t*‐test, and the Bayes factor indicated substantial evidence to accept the null hypothesis (Table 5, Figure 1b). The K–S test showed similar results (Table 3), and the distribution of voxel‐wise group median FA values for the CHIMERA‐adequate and deficient groups mostly overlapped (Figure 2b).

**TABLE 5:** One‐tailed Welch *t*‐test results for CHIMERA‐adequate FA > CHIMERA deficient FA

	Group	*Ex vivo*	*In vivo*
*N*	CHIMERA deficient	7	8
	CHIMERA‐adequate	11	9
Mean	CHIMERA deficient	0.313	0.272
	CHIMERA‐adequate	0.295	0.266
*t* value		−1.18	−1.00
Degrees of freedom		15.9	11.4
FDR‐adjusted *p* value		1.00	1.00
Hedge's *g* effect size (Adequate—Deficient)		−0.498	−0.475
Bayes factor		0.241	0.247

## 4 DISCUSSION

Our goal for this study was to investigate the effect of dietary intake of omega‐3 PUFAs in modulating the degree of white matter injury in mild TBI. We replicated previous results and showed a significant effect of the injury on FA in the optic tract and brachium of the superior colliculus, and the Bayes factor analysis indicated very strong and decisive evidence for higher FA values in sham animals compared to injured animals. Even though we detected the injury effect, we found no evidence of a neuroprotective effect of the diet adequate in omega‐3 PUFAs. The injured animals did not show a significant difference in FA between the adequate and deficient diet groups, and the Bayes factor analysis indicated substantial evidence to accept the null hypothesis and suggests that our results are not random or due to a sampling error. Moreover, the voxel‐wise FA distributions (Figure 2) of the two injured groups overlap, showing that the adequate diet had no effect on FA values following CHIMERA. Additionally, we were able to detect similar effects using both *ex vivo* and *in vivo* data, demonstrating that *in vivo* DTI is also sensitive to detecting the white matter damage resulting from CHIMERA.

### 4.1 Injury effect

Previous histological analyses indicated that two of the most damaged structures following CHIMERA were the optic tract and brachium of the superior colliculus (Haber et al., 2017; Namjoshi et al., 2014). These regions showed abundant silver staining suggesting disruption to the white matter. Staining with a pan‐neurofilament antibody revealed that this damage was in part a result of damaged axons, and IBA‐1 and GFAP staining showed increased glial activity after injury (Haber et al., 2017).

Haber et al. (2017) demonstrated that *ex vivo* diffusion MRI metrics, particularly FA, was extremely sensitive to detecting this white matter damage that resulted from CHIMERA. A subtraction map computed by subtracting the average sham FA image from the average CHIMERA FA image showed a reduction in FA for the CHIMERA group in the optic tract and brachium of the superior colliculus, the same regions shown by histology to be severely damaged. This is in line with previous work that has shown that reduced anisotropy can be linked with demyelination and axonal generation (Budde, Janes, Gold, Turtzo, & Frank, 2011), as well as increased number of glial cells (Saadani‐Makki et al., 2009).

In the current study, we were able to replicate the injury effect in FA, and clearly detected reduced FA in the optic tract and brachium of the superior colliculus in injured animals for both diet groups (Figure 4). We also observed similar results *in vivo* and showed that white matter injury in this model can be detected noninvasively by MRI. Remarkably, we still observed such a strong effect in both the *ex vivo* and *in vivo* data at a chronic time point of more than 3 months after injury, indicating that CHIMERA results in persistent disruption to the white matter.

### 4.2 Neuroprotective effect

While previous research has shown evidence for a neuroprotective of omega‐3 PUFAs on tissue damage following brain injury, our findings suggest that this effect may not be generalizable to all injury types. Much of the previous evidence for neuroprotection comes from work on ischemic models of brain injury. Ischemia results from restriction of cerebral blood flow that can deprive the brain tissue of oxygen and glucose, and can cause an accumulation of toxic substances that ultimately leads to cell death (Bramlett & Dietrich, 2004). Treatment with omega‐3 PUFAs both before and after ischemia results in reduced infarct volume and tissue damage, lower numbers of pro‐inflammatory glia, and a reduction in histological markers of cell death both at and near the infarct site (Belayev et al., 2011; Belayev, Khoutorova, Atkins, & Bazan, 2009; Cai et al., 2018; Fernandes et al., 2008; Hong, Belayev, Khoutorova, Obenaus, & Bazan, 2014; Jiang et al., 2016; Lalancette‐Hébert et al., 2011; Okada et al., 1996; Zhang et al., 2014, 2010).

TBI involves mechanical stresses on the tissue from an impact or acceleration and deceleration of the head. These mechanical effects can cause contusions and breakage of blood vessels that alter the cerebral blood flow to the brain tissue and result in effects similar to those observed in ischemia, but the mechanical stresses cause additional shearing and stretching of axons that lead to widespread white matter damage (Bramlett & Dietrich, 2004). A few studies have shown that omega‐3 PUFAs can decrease white matter damage by protecting myelin (Pu et al., 2013, 2017), and can reduce the markers of axonal damage such as APP and SMI‐32 (Bailes & Mills, 2010; Mills et al., 2010, 2011), but these studies were performed using injury models that are not biomechanically compatible with the type of injury most often seen in human TBI.

The current study applied CHIMERA as an injury model since it closely reproduces the type of white matter damage observed in human TBI and includes head rotation. In mice, CHIMERA reliably produces axonal damage and induces inflammatory effects in the optic tract and brachium of the superior colliculus (Haber et al., 2017; Namjoshi et al., 2014). This pattern of damage differs from the types investigated in the previous studies, which included a focal lesion (Pu et al., 2013, 2017), and strong damage to the corticospinal tract and medial lemniscus (Bailes & Mills, 2010; Mills et al., 2010, 2011). While our results do not refute those observed in these other injury models, they do bring into question the efficacy of omega‐3 PUFAs to provide neuroprotection against tissue damage following all types of injury, since it is possible that the biomechanical differences between CHIMERA and these other models result in different pathophysiological effects, or that damage to cerebral white matter is not as accessible or responsive to omega‐3 PUFAs as tissue near a lesion or in the brainstem. Future work might examine differences in omega‐3 PUFA neuroprotection between models more closely to determine how the omega‐3 PUFAs might best be translated to the study of human TBI.

Additionally, the findings of the current study may depart from those in previous studies that show a robust neuroprotective effect of omega‐3 PUFAs because we investigated neuroprotective effects of the adequate diet at a chronic time point of more than 3 months postinjury. Most of the previous studies limited the postinjury time period to 7–35 days (Trépanier et al., 2016), and only two studies examined neuroprotective effects at 8 weeks (Cai et al., 2018; Fernandes et al., 2008). Investigating the efficacy of a treatment at a more chronic time point is especially important for translational studies of TBI, since even mild TBI is capable of producing long‐lasting effects in the human brain that can worsen with time (Corrigan & Hammond, 2013).

For any treatment, or in this case, diet, to be considered effective, the beneficial effects should be detectable at a chronic time point in regions with remaining tissue damage. In this case, we did not detect any beneficial effects from the diet and found a paradoxical effect of reduced FA in the injured adequate diet group, suggesting that the adequate diet did not provide neuroprotection from injury. The possibility remains that the dietary effects may have been more easily observable in the acute period and may have resulted in a faster recovery in animals with the adequate diet. However, even if the diet was to confer such benefits in the acute period after injury, the lack of long‐term effects indicates that this type of dietary intervention may not be helpful for protecting brain tissue following a type of injury known to have so many chronic effects. To characterize the potential neuroprotective effect across the entire recovery period, future work might employ a longitudinal study design that uses multiple time points within the same animal to distinguish between neuroprotection in the acute and chronic phases postinjury and also reduce variability to better detect subtle changes due to the diet.

### 4.3 *Ex vivo* and *in vivo* imaging results

*In vivo* MRI can be performed noninvasively and is an essential tool for translating findings from animal models into research and applications for human patients. *In vivo* imaging, however, can be limited by reduced SNR and increased image artifacts compared to *ex vivo* imaging (Sadeghi et al., 2015; Walker et al., 2011). While many of these issues can be corrected in processing, *in vivo* imaging may still suffer from lower resolution due to a shorter allowable scan time that may result in partial volume effects that reduce the ability to detect more subtle microstructural differences. It is reassuring to see that *in vivo* imaging is as sensitive to detecting white matter injury resulting from CHIMERA as *ex vivo* imaging.

The *in vivo* diffusion MRI results from the current study replicate the results that have been found using *ex vivo* data. We have shown that *in vivo* diffusion MRI is capable of detecting white matter damage and can be useful in detecting such effects resulting from TBI. Our results from *in vivo* MRI are also particularly encouraging for the study of white matter damage in human patients suffering from persistent effects of TBI since we were still able to detect these effects in the chronic period following the injury.

## 5 CONCLUSIONS

In summary, in the current study, we applied both *ex vivo* and *in vivo* quantitative diffusion MRI to investigate a potential neuroprotective effect of omega‐3 PUFAs on mild TBI. We replicated the effects of CHIMERA that had been previously observed with histology *ex vivo* DTI and histology with both sets of data. While we clearly replicated *ex vivo* and *in vivo* the detection of significant injury effects for FA in the optic tract and brachium of the superior colliculus that were shown in previous studies, our results also indicate that omega‐3 PUFAs do not offer a neuroprotective effect on white matter injury resulting from mild TBI in the CHIMERA model. Because we focused on the chronic phase greater than 3 months after the injury, further investigation is necessary to investigate potential neuroprotective effects of omega‐3 PUFAs longitudinally to fully characterize effects throughout the acute and chronic phases postinjury and eliminate intersubject variability to detect more subtle microstructural differences. Future work might also compare neuroprotective effects of omega‐3 PUFAs in different injury models to assess their applicability to human TBI.

## DECLARATION OF TRANSPARENCY

The authors, reviewers and editors affirm that in accordance to the policies set by the *Journal of Neuroscience Research*, this manuscript presents an accurate and transparent account of the study being reported and that all critical details describing the methods and results are present.

## CONFLICT OF INTEREST

No potential conflict of interest was reported by the authors.

## AUTHOR CONTRIBUTIONS

*Conceptualization,* H.Y.K. and C.P.; *Methodology,* A.D., H.C., A.B., A.K., B.D., H.Y.K., and C.P.; *Investigation,* L.D.R.; *Validation,* T.H.; *Formal Analysis,* L.D.R. and T.H.; *Resources,* A.D., H.C., A.B., A.K., and B.D.; *Writing – Original Draft,* L.D.R.; *Writing – Review & Editing,* L.D.R., A.D., T.H., B.D., H.Y.K., and C.P.; *Visualization,* L.D.R.; *Supervision,* C.P.; *Funding Acquisition,* H.Y.K. and C.P.

### PEER REVIEW

The peer review history for this article is available at https://publons.com/publon/10.1002/jnr.24705.

## Supporting information

Transparent Peer Review Report

Transparent Science Questionnaire for Authors

## Data Availability

The data that support the findings of this study are available from the corresponding author upon reasonable request.
