# Supplementary material for: Investigation of the effect of dietary intake of omega‐3 polyunsaturated fatty acids on trauma‐induced white matter injury with quantitative diffusion MRI in mice
Source: J Neurosci Res. 2020 Aug 25;98(11):2232–44. doi: 10.1002/jnr.24705 (PMC7589213; doi:10.1002/jnr.24705)
Supplement: Supplementary file 1 — Transparent Peer Review Report [file JNR-98-2232-s001.docx]

**Investigation of the effect of dietary intake of omega-3 polyunsaturated fatty acids on trauma induced white matter injury with quantitative diffusion MRI in mice**

Laura D. Reyesa,b, Thaddeus Haightb,c, Abhishek Desaid , Huazhen Chend , Asamoah Bosomtwib,e,f , Alexandru Korotcovb,f, Bernard Dardzinskif , Hee-Yong Kimc , Carlo Pierpaolia

_____________________________________________________________________________________

| Review timeline: | Submission date: 04-Mar-2020  Editorial Decision: Minor Modification (12-May-2020)  Revision Received: 07-Jun-2020  Editorial Decision: Accept with Minor Edits (30-Jun-2020)  Revision Received: 09-Jul-2020  Editorial Decision: Accept (ACC) (11-Jul-2020)  Accepted:11-Jul-2020 |  |
| --- | --- | --- |
|  |  |  |

Editor:	Howland, Dena

Reviewer 1: Badea, Alexandra

Reviewer 2: Thompson, Floyd

_____________________________________________________________________________________

1st Editorial Decision	12-May-2020

____________________________________________________________________________________

Dear Dr Reyes:

Thank you for submitting your manuscript to the Journal of Neuroscience Research. We have now received the reviewer feedback and have appended those reviews below. I am glad to say that the reviewers are overall very enthusiastic and supportive of the study. They did raise some concerns and made some suggestions for clarification, but I expect that these points should be relatively straightforward to address. If there are any questions or points that are problematic, please feel free to contact me. I will be glad to discuss.

We ask that you return your manuscript within 30 days. Please explain in your cover letter how you have changed the present version. If you require longer than 30 days to make the revisions, please contact Dr Junie Warrington (jpwarrington@umc.edu). You can submit your revised manuscript directly by clicking on the following link: *** PLEASE NOTE: This is a two-step process. After clicking on the link, you will be directed to a webpage to confirm. ***

https://mc.manuscriptcentral.com/jnr?URL_MASK=28e2ad7a61f0479e932ba163dcdd82fa

Thank you again for your submission to the Journal of Neuroscience Research; we look forward to reading your revised manuscript.

Best Wishes,

Dr Dena Howland
Associate Editor, Journal of Neuroscience Research

Dr Junie Warrington
Editor-in-Chief, Journal of Neuroscience Research

Editorial Comments to Author:
1. Please ensure that tables and figures are included at the end of the manuscript, after the references. Additionally, Figure Legends should be listed together after the reference section.

2. Please upload a graphical abstract, which we are asking of all authors submitting original research articles. This is intended to provide readers with a visual representation of the conclusions and an additional way to access the contents and appreciate the main message of the work. What we require is a .tif image file and a .doc text file containing an abbreviated abstract. For the image, labels, although useful, must be kept to a minimum and the image should be 400 x 300, 300 x 400, or 400 x 400 pixels square and at a resolution of 72 dpi. This can be one of the figures from your article, or something slightly different, as long as it represents your study.  Instructions for this can be found in our author guidelines online at http://onlinelibrary.wiley.com/journal/10.1002/(ISSN)1097-4547/homepage/ForAuthors.html


Associate Editor: Howland, Dena
Comments to the Author:
Please address the issues raised by Reviewer 1 and the Statistical Editor.  These revisions are considered minor.

Statistics Editor: McArthur, David
Comments to the Author:
A commendable study from a statistical viewpoint!  This reviewer has only minor issues:

Figure 1 shows dotplots with substantial numbers of those dots overplotted atop one another.  An easy recrafting of these plots is with a slight jitter along the x-axis to avoid such overplotting, or instead to depict with boxplots or violinplots.

Figure 1's caption indicates "they [sic] Bayes factor indicated substantial evidence" but the actual value is not shown.  The caption ends with "Bayes factor analysis suggested" but again without showing the actual value or values.

Figure 2 shows horizontal gold lines in both A and B that seem to be in the same color as "CHIMERA deficient" yet that curve is clearly visible.  Those horizontal lines in gold should be omitted unless they have some meaning not yet explained.

Figures 3 and 4 present grey brain imaging against uniformly grey backgrounds.  Such images can be problematic for readers with any sort of visual impairment, and there would be no loss of meaning whatseover to replace the background with white and change white lettering to black (except where letters are placed against the grey brain imaging).  

The Avants reference provides no access information.  This is also true for both the Morey & Rouder reference and the Wickham reference.  The R reference should include the citation as shown in citation() and the version number.

Reviewer: 1

Comments to the Author
Investigation of the effect of dietary  intake of omega-3 ployunsaturated fatty acids on trauma induced white matter injury with quantitative diffusion MRI in mice

This is a well written and executed study that assesses using C57BL6 mouse models both the effects of a closed head injury model through engineered rotational acceleration, and the proposed neuroprotective effects of a diet enriched in imega-3 polyunsaturated fatty acids on white matter tract integrity. While the methods employed were able to detect an effect of injury, confirming previous findings, they were not able to detect a neuroprotective effect of diet.  

Major comments/suggestions
I wonder if the deformation fields could be used to detect volume changes, and if these effects may not be larger than the FA effects.
Since the study used a multishell acquisitions, is it possible that other metric beyond FA could have been obtained, and show more sensitivity, e.g. from SHORE? Also were other DTI metric explored , such as RD or ADC?

Minor comments
Methods:
1. What was the gradient strength on the system used?
2. What was the duration of the scans
3. Would it be more consistent to stick to non parametric tests throughout, or why choose Welch in the beginning, then Kolmogorov-Smirnoff afterwards.
Results

The first subtitle refers to ex vivo analyses, but then the results are presented at the same time for in vivo and ex vivo statistics. I suggest removing the subtitle.

I suggest you may include the Cohen effect size in the results (tables).

Figure 1 could use a box plot, for an easier visual interpretation.

Legend of figure 1 , line 50 “they Bayes factor” -> “the Bayes factor”.

Table 3 – Please define D.

Figure 2 legend ; it may help to remind the reader that she is looking at one particular region.

A color scale (not just gray) may be helpful in Figure 3; the signed differences are hard to interpret just using a grayscale.


Reviewer: 2

Comments to the Author
Well designed and comprehensive study.

_____________________________________________________________________________________

Authors’ Response

Dear Dr. Howland and Dr. Warrington,

Thank you for considering our manuscript. We also thank the reviewers for their thorough assessment of our work. We have revised our manuscript based on their feedback and our specific responses are outlined below. We hope we have addressed all concerns adequately.

Best regards,

Laura Reyes

Editorial Comments to Author:
Please ensure that tables and figures are included at the end of the manuscript, after the references. Additionally, Figure Legends should be listed together after the reference section.
• The figure captions are now included immediately after the references, followed by the figures and tables.

Please upload a graphical abstract, which we are asking of all authors submitting original research articles. This is intended to provide readers with a visual representation of the conclusions and an additional way to access the contents and appreciate the main message of the work. What we require is a .tif image file and a .doc text file containing an abbreviated abstract. For the image, labels, although useful, must be kept to a minimum and the image should be 400 x 300, 300 x 400, or 400 x 400 pixels square and at a resolution of 72 dpi. This can be one of the figures from your article, or something slightly different, as long as it represents your study. Instructions for this can be found in our author guidelines online at http://onlinelibrary.wiley.com/journal/10.1002/(ISSN)1097-4547/homepage/ForAuthors.html
• We have uploaded a graphical abstract.

Statistics Editor:
Comments to the Author:
A commendable study from a statistical viewpoint!
• We put a lot of effort in the design of the experiment and we are grateful to the reviewer for his/her comment.

This reviewer has only minor issues:
Figure 1 shows dotplots with substantial numbers of those dots overplotted atop one another. An easy recrafting of these plots is with a slight jitter along the x-axis to avoid such overplotting, or instead to depict with boxplots or violinplots.
• We made new versions of these plots with a violin plot to show the distribution of the data as suggested. In addition, In the revised plots the mean is indicated by a dot with bars representing the standard deviation.

Figure 1's caption indicates "they [sic] Bayes factor indicated substantial evidence" but the actual value is not shown. The caption ends with "Bayes factor analysis suggested" but again without showing the actual value or values.
• We corrected the misspelled word in the Figure 1 caption. We also added the Bayes factor values to the caption.

Figure 2 shows horizontal gold lines in both A and B that seem to be in the same color as "CHIMERA deficient" yet that curve is clearly visible. Those horizontal lines in gold should be omitted unless they have some meaning not yet explained.
• We agree that these gold lines did not contribute to the clarity of the plot and we removed them.

Figures 3 and 4 present grey brain imaging against uniformly grey backgrounds. Such images can be problematic for readers with any sort of visual impairment, and there would be no loss of meaning whatseover to replace the background with white and change white lettering to black (except where letters are placed against the grey brain imaging).
• Figure 3 and 4 were both changed to have black text on a white background surrounding the images. The text that was placed against the gray background within or near the images was also changed to become easier to read, and is now black on a white background.

The Avants reference provides no access information. This is also true for both the Morey & Rouder reference and the Wickham reference. The R reference should include the citation as shown in citation() and the version number.
• These three references have been updated to include links on how to access the packages.


Reviewer: 1
Comments to the Author:
Investigation of the effect of dietary intake of omega-3 polyunsaturated fatty acids on trauma induced white matter injury with quantitative diffusion MRI in mice

This is a well written and executed study that assesses using C57BL6 mouse models both the effects of a closed head injury model through engineered rotational acceleration, and the proposed neuroprotective effects of a diet enriched in omega-3 polyunsaturated fatty acids on white matter tract integrity. While the methods employed were able to detect an effect of injury, confirming previous findings, they were not able to detect a neuroprotective effect of diet.
• This is indeed the main finding of our study. We are grateful to the reviewer for his/her positive comments on our study.

Major comments/suggestions
I wonder if the deformation fields could be used to detect volume changes, and if these effects may not be larger than the FA effects. Since the study used a multishell acquisitions, is it possible that other metric beyond FA could have been obtained, and show more sensitivity, e.g. from SHORE? Also were other DTI metric explored, such as RD or ADC?

• We find the suggestions of using additional metrics from a higher order model (e.g. propagator anisotropy (PA) or non-gaussianity from a MAPMRI fitting) or a diffusion-driven tensor based morphometry (D-TBM) approach interesting and we agree with the reviewer that these metrics may be useful in examining the injury and potential neuroprotective effects. However, by using these metrics our study will became exploratory in nature, as these metrics have not been validated as biomarkers for white matter damage in this model. In designing this hypothesis driven study, we relied on FA as a metric to assess white matter damage because a previous paper (Haber et al. 2017) validated the use of FA as a biomarker with specificity and sensitivity comparable to that of histology in this injury model. This previous study found that diffusivity measures (Trace, AD, RD) were not as sensitive as FA to the injury and did not investigate any MAP-MRI or DTBM metrics. Nevertheless we find the reviewer suggestions intriguing for further “model characterization” analyses. We intend to pursue a future study with whole brain survey of these metrics along with histology to validate their potential use as biomarkers of tissue damage in CHIMERA.

Minor comments
Methods:
1. What was the gradient strength on the system used?
• We have clarified in the methods section that the acquisitions were performed on a 7T small animal (20 cm horizontal bore) Bruker BioSpec scanner (Billerica, MA, USA) with 120 mm diameter, 670 mT/m gradient coils using the ParaVision 6.0.1 software.

2. What was the duration of the scans
• We have added that the total in vivo scan time for each animal was one hour and 45 minutes, while the total ex vivo scan time was 15 hours and 11 minutes.

3. Would it be more consistent to stick to non parametric tests throughout, or why choose Welch in the beginning, then Kolmogorov-Smirnoff afterwards.
• We thank the reviewer for the comment. The Welch t-test (or unequal variances t-test), which is a more robust test of samples with unequal variances and/or non-overlapping distributions, was used to examine differences in group means based on distributions of individuals’ median FA values (See Figure 1). These median FA values had a distribution pattern for which the mean was an appropriate measure of central tendency for each of the groups which we wished to compare (e.g., sham deficient vs. sham adequate).

The K-S tests were based on distributions of FA representative of the ROI in each diet and injury group (i.e. sham deficient, sham adequate, CHIMERA deficient, CHIMERA adequate). This distribution was previously described in the Statistical analysis section:

In our previous analyses, we performed tests using the median FA value within the optic tract and brachium ROI from each animal. While these median values are capable of showing the central tendency of the FA values in the ROI, they may not properly describe potential differences in the tails of the distribution of FA values in the ROI.

Given that the distribution of FA values is skewed within the ROI, the Kolmogorov-Smirnoff test was performed to account for the remote possibility that white matter damage would have affected preferentially the highest FA values (tail of the distribution within the ROI) and not the median of the distribution. The results of this analysis exclude this possibility quite convincingly (See Figure 2).

Results
The first subtitle refers to ex vivo analyses, but then the results are presented at the same time for in vivo and ex vivo statistics. I suggest removing the subtitle.
• This was an excellent suggestion as it streamlined our results section. We have removed the separation between ex vivo and in vivo results as suggested, and they are now discussed in the same sections.

I suggest you may include the Cohen effect size in the results (tables).
• We computed Hedge’s g, a similar effect size metric to Cohen’s d that uses pooled standard deviation weighted by the sample size of each group, and included these values in the appropriate tables and also in text.

Figure 1 could use a box plot, for an easier visual interpretation.
• As suggested and addressed previously, we made new versions of these plots with a violin plot to show the distribution of the data. In addition, In the revised plots the mean is indicated by a dot with bars representing the standard deviation.
•

Legend of figure 1, line 50 “they Bayes factor” -> “the Bayes factor”.
• We corrected the misspelled word in the Figure 1 caption.

Table 3 – Please define D.
• D is now defined in a caption for Table 3.

Figure 2 legend; it may help to remind the reader that she is looking at one particular region.
• The figure 2 caption now specifies that the distributions shown are from the optic tract and brachium ROI.

A color scale (not just gray) may be helpful in Figure 3; the signed differences are hard to interpret just using a grayscale.
• After consideration and viewing of different color scales with our results, we have decided to keep the gray scale map since it allows for the viewer to fully appreciate the magnitude of the effect size differences (for example, the extremely dark regions in Figure 4) without biasing the interpretation with the color selection and avoiding difficulty in interpreting intermediate colors created when using a color scale. The signed differences in the images in Figure 3 appear difficult to interpret because the values in the maps are relatively flat and have no apparent patterns or clusters. The flatness of these maps shows that there is little or no effect of the diet in these regions.

Reviewer: 2
Comments to the Author:
Well designed and comprehensive study.
• Thank you for the positive comment.

_____________________________________________________________________________________

2^nd^ Editorial Decision

30-Jun-2020

Dear Dr Reyes:

Thank you for submitting your manuscript to the Journal of Neuroscience Research. We have now received the reviewer feedback and have appended those reviews below. I am glad to say that the reviewers are overall very enthusiastic and supportive of the study. They did raise some minor concerns and made some suggestions for clarification, but I expect that these points should be relatively straightforward to address. If there are any questions or points that are problematic, please feel free to contact me. I will be glad to discuss.

We ask that you return your manuscript within 15 days. Please explain in your cover letter how you have changed the present version. If you require longer than 15 days to make the revisions, please contact Dr Junie Warrington (jpwarrington@umc.edu).You can submit your revised manuscript directly by clicking on the following link: *** PLEASE NOTE: This is a two-step process. After clicking on the link, you will be directed to a webpage to confirm. ***

https://mc.manuscriptcentral.com/jnr?URL_MASK=0dc51943d59346a0be99544e021d7db3

Thank you again for your submission to the Journal of Neuroscience Research; we look forward to reading your revised manuscript.

Best Wishes,

Dr Dena Howland
Associate Editor, Journal of Neuroscience Research

Dr Junie Warrington
Editor-in-Chief, Journal of Neuroscience Research

Editorial Comment to Authors:
- Please note that the graphical abstract should summarize the main message of the study in graphical form. We ask that you update the graphical abstract so that the main message is clear. Also, we ask that you increase the resolution of the image as well.
- Please also include individual data points with the violin plots, as suggested by the associate editor.


Associate Editor: Howland, Dena
Comments to the Author:
We appreciate that all comments have been addressed by the authros.  Acceptance for publication is conditional based upon three remaining minor, but critical corrections for publication. These are:

1.  Plot all data points within the violin plots, jitter these points and use a different symbol for the mean point (e.g. diamonds for the individual points and a circle for the mean).  We realize that the language in the initial review could have been interpreted to mean either violin plots or jittering points was acceptance.  The plotting of all data is required for data transparency.  

2. Reduce the graphical abstract text to 50 or fewer words.  It currently is at 77 words.   Please increase the text size on the graphical abstract figure so that it is readable in a small format print size and increase the resolution of the graphical abstract figure to a minimum of 72 dpi (currently is 40 dpi).

We look forward to receiving the revised graphical abstract figure/text along with a revised Figure 1 (violin plots).

Reviewer: 2

Comments to the Author
Comprehensive report of your well designed study. Your manuscript reports important data regarding the potential of diet supplementation using omega-3 polyunsaturated fatty acids for neuroprotection following a clinically relevant TBI.

Reviewer: 1

Comments to the Author
Thank you for the responses, please double check if the violin plots show the mean with the dot, or the median.

_____________________________________________________________________________________

Authors’ Response

Dear Dr. Howland and Dr. Warrington,

Thank you for these additional comments to improve our manuscript. We also thank the reviewers for their assessment of our work. We have revised our manuscript based on the recent feedback and the responses are given below.

Best regards,

Dr. Laura Reyes

Editorial Comment to Authors:
- Please note that the graphical abstract should summarize the main message of the study in graphical form. We ask that you update the graphical abstract so that the main message is clear. Also, we ask that you increase the resolution of the image as well.
- Please also include individual data points with the violin plots, as suggested by the associate editor.


Associate Editor: Howland, Dena
Comments to the Author:
We appreciate that all comments have been addressed by the authors. Acceptance for publication is conditional based upon three remaining minor, but critical corrections for publication. These are:

1. Plot all data points within the violin plots, jitter these points and use a different symbol for the mean point (e.g. diamonds for the individual points and a circle for the mean). We realize that the language in the initial review could have been interpreted to mean either violin plots or jittering points was acceptance. The plotting of all data is required for data transparency.
• We have revised the plots to show violin plots, the mean with a solid diamond, standard deviation lines, and individual data points with partially transparent circles that have been jittered to reduce overlap.

2. Reduce the graphical abstract text to 50 or fewer words. It currently is at 77 words. Please increase the text size on the graphical abstract figure so that it is readable in a small format print size and increase the resolution of the graphical abstract figure to a minimum of 72 dpi (currently is 40 dpi).
• We reduced the length of the graphical abstract to 50 words, and we have refined the text to clarify the main message.
• The text size in the graphical abstract figure was enlarged compared to the previous submission, and the resolution of the graphical abstract has been increased to 72 dpi.

We look forward to receiving the revised graphical abstract figure/text along with a revised Figure 1 (violin plots).

Reviewer: 2

Comments to the Author
Comprehensive report of your well designed study. Your manuscript reports important data regarding the potential of diet supplementation using omega-3 polyunsaturated fatty acids for neuroprotection following a clinically relevant TBI.
• Thank you for the positive comment.

Reviewer: 1

Comments to the Author
Thank you for the responses, please double check if the violin plots show the mean with the dot, or the median.
• The values in the plots have been checked, and the dots (now diamonds) show the mean, and the lines show the standard deviation.

_____________________________________________________________________________________

3^rd^ Editorial Decision

11-Jul-2020

Dear Dr Reyes:

Thank you for submitting your manuscript "Investigation of the effect of dietary intake of omega-3 polyunsaturated fatty acids on trauma induced white matter injury with quantitative diffusion MRI in mice" by Reyes, Laura; Haight, Thaddeus; Desai, Abhishek; Chen, Huazen; Bosomtwi, Asamoah; Korotcov, Alexandru; Dardzinski, Bernard; Kim, Hee-Yong; Pierpaoli, C.

You will be pleased to know that your manuscript has been accepted for publication. Thank you for submitting this excellent work to our journal.

In the coming weeks, the Production Department will contact you regarding a copyright transfer agreement and they will then send an electronic proof file of your article to you for your review and approval.

Please note that your article cannot be published until the publisher has received the appropriate signed license agreement. Within the next few days, the corresponding author will receive an email from Wiley’s Author Services asking them to log in. There, they will be presented with the appropriate license for completion. Additional information can be found at https://authorservices.wiley.com/author-resources/Journal-Authors/licensing-open-access/index.html

Congratulations on your results, and thank you for choosing the Journal of Neuroscience Research for publishing your work. I hope you will consider us for the publication of your future manuscripts.

Sincerely,

Dr Dena Howland
Associate Editor, Journal of Neuroscience Research

Dr Junie Warrington
Editor-in-Chief, Journal of Neuroscience Research


Associate Editor: Howland, Dena
Comments to the Author:
Thank you for making the requested edits.  There are no further revisions needed and we are pleased to accept your manuscript for publication in the Journal of Neuroscience Research.


NIH GRANTEES: All NIH authors should also be aware that Wiley-Blackwell will submit manuscripts to the NIH Manuscript Submission system automatically. After their accepted manuscript files have been posted by Wiley-Blackwell to the NIH Manuscript Submission system, the corresponding author will receive an e-mail request from PubMed Central asking them to approve the upload for display on the PubMed Central system. Authors will need to provide approval to complete the process of deposition to PubMed Central. This is a requirement of their grant/affiliation.

P.S. You can help your research get the attention it deserves! Check out Wiley’s Promotion Guide for best-practice recommendations for promoting your work at www.wileyauthors.com/maximize.

_____________________________________________________________________________________
